# OmicsSuite: a customized and pipelined suite for analysis and visualization of multi-omics big data

**DOI:** 10.1093/hr/uhad195

**Published:** 2023-09-28

**Authors:** Ben-Ben Miao, Wei Dong, Yi-Xin Gu, Zhao-Fang Han, Xuan Luo, Cai-Huan Ke, Wei-Wei You

**Affiliations:** State Key Laboratory of Marine Environmental Science, College of Ocean and Earth Sciences, Xiamen University, Xiamen 361102, Fujian, China; Hospital of Stomatology, Guanghua School of Stomatology, Guangdong Provincial Key Laboratory of Stomatology, Sun Yat-Sen University, Guangzhou 510055, Guangdong, China; College of Fisheries, Guangdong Ocean University, Zhanjiang 524088, Guangdong, China; State Key Laboratory of Marine Environmental Science, College of Ocean and Earth Sciences, Xiamen University, Xiamen 361102, Fujian, China; State Key Laboratory of Marine Environmental Science, College of Ocean and Earth Sciences, Xiamen University, Xiamen 361102, Fujian, China; State Key Laboratory of Marine Environmental Science, College of Ocean and Earth Sciences, Xiamen University, Xiamen 361102, Fujian, China; State Key Laboratory of Marine Environmental Science, College of Ocean and Earth Sciences, Xiamen University, Xiamen 361102, Fujian, China; Fujian Institute for Sustainable Oceans, Xiamen University, Xiamen 361102, Fujian, China

## Abstract

With the advancements in high-throughput sequencing technologies such as Illumina, PacBio, and 10X Genomics platforms, and gas/liquid chromatography-mass spectrometry, large volumes of biological data in multiple formats can now be obtained through multi-omics analysis. Bioinformatics is constantly evolving and seeking breakthroughs to solve multi-omics problems; however, it is challenging for most experimental biologists to analyse data using command-line interfaces, coding, and scripting. Based on experience with multi-omics, we have developed OmicsSuite, a desktop suite that comprehensively integrates statistics and multi-omics analysis and visualization. The suite has 175 sub-applications in 12 categories, including Sequence, Statistics, Algorithm, Genomics, Transcriptomics, Enrichment, Proteomics, Metabolomics, Clinical, Microorganism, Single Cell, and Table Operation. We created the user interface with Sequence View, Table View, and intelligent components based on JavaFX and the popular Shiny framework. The multi-omics analysis functions were developed based on BioJava and 300+ packages provided by the R CRAN and Bioconductor communities, and it encompasses over 3000 adjustable parameter interfaces. OmicsSuite can directly read multi-omics raw data in FastA, FastQ, Mutation Annotation Format, mzML, Matrix, and HDF5 formats, and the programs emphasize data transfer directions and pipeline analysis functions. OmicsSuite can produce pre-publication images and tables, allowing users to focus on biological aspects. OmicsSuite offers multi-omics step-by-step workflows that can be easily applied to horticultural plant breeding and molecular mechanism studies in plants. It enables researchers to freely explore the molecular information contained in multi-omics big data (Source: https://github.com/OmicsSuite/, Website: https://omicssuite.github.io, v1.3.9).

## Introduction

Over the past decade, the widespread application of next-generation sequencing (NGS) technologies represented by Illumina Solexa and HiSeq platforms [1], as well as third-generation sequencing (TGS) technologies led by PacBio Sequel [2] and Oxford Nanopore [3] platforms, has revolutionized the fields of molecular, evolutionary, and computational biology. Currently, omics diversity and integrated multi-omics analysis are thriving research fields, and multidimensional analysis of biological features and mechanisms has become more precise and efficient [4]. With the widespread application of sequencing and mass spectrometry technologies, the quantity and storage types of data generated are also rapidly increasing [5]. For example, data generated by PacBio HiFi sequencing has longer reads and higher throughput, and storage formats based on GC/LC–MS (Gas/Liquid Chromatography Mass Spectrometry) such as mzXML (mass spectrometric data in eXtensible Markup Language), mzData (mass spectrometric Data), and mzML [6] are gradually evolving to meet more diverse needs. At the same time, data from 10X Genomics [7], ranging from Chromium matrix data generated from single-cell transcriptomics to Visium HDF5 (Hierarchical Data Format version 5) data generated from spatial transcriptomics, have become more complex and mysterious. As a result, there are significant differences in the analytical pipelines, methods, and programs employed in data parsing for downstream bioinformatic analysis across genomics, transcriptomics, proteomics, metabolomics, microbial omics, and single-cell omics [8].

**Figure 1:**
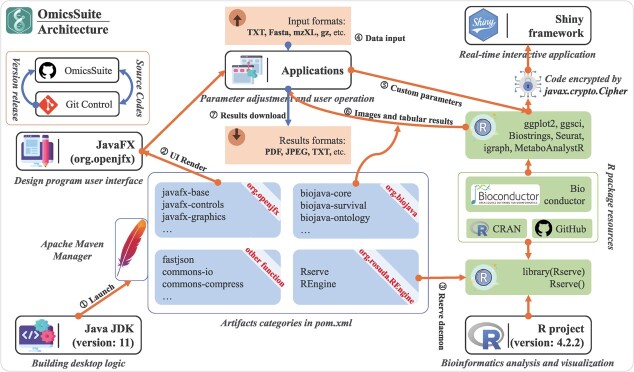
The OmicsSuite native innovation framework architecture and the integration of the Posit Shiny framework. The arrows show execution logical steps, dataset inputs and results outputs.

Omics is used in molecular biology, while bioinformatics employs methods from mathematics, informatics, statistics, and computer science to analyse biological problems [9]. With the rapid development of sequencing and mass spectrometry technologies and innovation in bioinformatics, programs based on command line or integrated installation and online analysis services are constantly emerging. This has also benefited from the rapid development of internet and cloud technology, the efficient development frameworks, and the contributions of developers [10]. Online analysis services facilitate researchers’ access to programs and submission of tasks; for example, EMBL (https://www.ebi.ac.uk/services) provides many services for nucleotide sequence analysis, including Clustal Omega multi-sequence alignment [11]. ExPASy (Expert Protein Analysis System) (https://www.expasy.org) implements a series of amino acid sequence analysis services, including SWISS-MODEL protein structure prediction [12]. Open-source Python plugins based on the Bio-Galaxy cloud framework (https://github.com/galaxyproject/galaxy) provide a mature self-built biological cloud platform [13], and the HiPlot platform provides free and convenient online visualization applications for researchers [10]. Online cloud platforms often require powerful computational resources for support and require professional internet teams to assist in development and maintenance. At the same time, omics data often has the characteristics of large volumes and complex analysis steps, making online services more suitable for lightweight analysis focused on a single process. The Python-based BioConda (https://github.com/bioconda/) community [14], the R-based CRAN (Comprehensive R Archive Network) (https://cran.r-project.org) [15], the Bioconductor (https://www.bioconductor.org) [16] community as well as BioPerl [17] and BioJava [18] are particularly active in the field of bioinformatics. Most of these programs are used in command-line form, for example BioPython [19] for sequence and structure analysis based on Python, BWA for short reads alignment based on Clang [20], Trimmomatic for FastQ raw data filtering based on Java [21], and Trinity for RNA-Seq de-novo assembly based on Perl [22]. Python, R, and Perl codes that run as scripts have powerful features. Command-line or script-based programs have a high learning curve, making them better suited for senior developers. As a result, desktop programs are more suitable for situations where large computing resources and development are not required, compared to online servers. Such programs are also more user-friendly and stable than command-line interfaces. Examples include MEGA (Molecular Evolutionary Genetics Analysis) for multiple sequence alignment and phylogenetic tree construction [23], BioEdit (https://thalljiscience.github.io) for sequence editing and manipulation, and TBtools [24] (Toolkit for Biologists integrating tools) for sequence processing and various forms of visualization. Although these programs have been well received, there is currently no suite of programs that integrates bioinformatics and multivariate analysis.

Based on our experience analysing multi-omics data, we developed a stable desktop suite called OmicsSuite that provides interactive and user-friendly multi-omics analysis and visualization. The suite currently comprises 175 sub-applications, covering various data analysis and visualization tasks. OmcisSuite is suitable for conducting plant chloroplast genome analysis, exploring breeding-related variation loci through GWAS, deciphering gene expression mechanisms through transcriptomic analysis, uncovering compounds related to plant color and taste through metabolomic analysis, and providing the latest single-cell RNA-Seq and spatial transcriptomic analysis pipelines. OmicsSuite offers multi-omics step-by-step workflows that can be easily applied to horticultural plant breeding and molecular mechanism studies in plants.

## Results and discussions

### OmicsSuite native framework architecture

OmicsSuite is an innovative framework for analysing and visualizing multi-omics data in a workflow (Fig. 1). The JavaFX library provides user interface (UI) control methods, parameter component classes, web engine support, and other interface display and friendly interaction functions through a series of sub-libraries such as javafx-controls, javafx-graphics, and javafx-web. The interfaces and analysis parameters of all 175 sub-applications are implemented through various components provided by JavaFX. Each sub-application provides essential interfaces such as uploading example datasets or user data files, parameter synchronization and feedback, and outputting results. Rserve and REngine provided by org.rosuda.REngine (https://github.com/s-u/REngine/) as two special and critical libraries are used to implement real-time communication with the R environment through daemon threads. Correspondingly, the Rserve function is utilized to provide instant responses to Java call signals.

BioJava is the exclusive library for Java in the field of bioinformatics, consisting of a series of analysis functions applied to data from base sequences to molecular structures. Several applications benefit from the development of biojava-core, biojava-survival, biojava-ontology, and other sub-libraries provided by BioJava. Most of the functions of sequence analysis, statistics and algorithms, multi-omics analysis, and visualization in OmicsSuite are provided by 300+ open-source packages covering a wide range of fields and subjects. These packages are provided by the Bioconductor and CRAN communities, as well as the GitHub open-source code platform. OmicsSuite selects the most suitable packages for implementing various functions, including visualization functions and custom embellishment components based on ggplot2 [25], prioritization of color palettes provided by ggsci (https://github.com/cran/ggsci/), building molecular regulatory networks based on igraph [26], using Biostrings [27] to standardize basic sequence types, and integrating Seurat v4 [28] and Monocel2 [29] for single-cell data analysis. Furthermore, the Shiny framework, which benefits from the support of the R system and modern interactive user interfaces, has contributed to many high-quality online analysis services [30]. OmicsSuite has independently developed 20+ Shiny applications for users to choose from. OmicsSuite provides exclusive service control functions for the Shiny framework, enabling users to start and stop Shiny services at any time. The applications provide examples and allow uploads of large files.

Although OmicsSuite is used by Java and R as the real-time running environment and function execution environment, respectively, and has a large number of built-in Java and R modules, and even the Shinyapp framework, users only need to install based on binary files to use the full functionality out of the box, without any additional configuration environment and dependencies. Basically, a computer device with a 4-cores CPU, 4G memory, and 256G storage can perform normal operations of OmicsSuite. We recommend providing a minimum of 6-cores CPU and 8GB memory for single-cell analysis, with a test Peripheral Blood Mononuclear Cells (PBMC) dataset (including 2700 single cells) execution time of approximately 3 minutes.

### UI design and data interface

**Figure 2:**
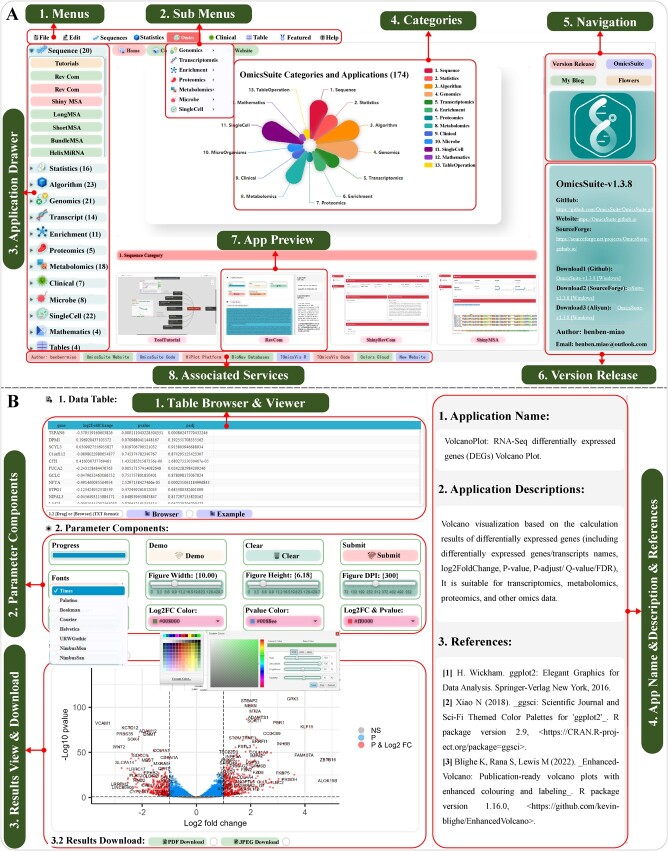
OmicsSuite layout and sub-application user interface. **(A)** The layout of OmicsSuite; **(B)** The sub-application user interface is divided into three sections from top to bottom: data upload and preview, parameter components, and results preview and download.

OmicsSuite has redesigned the UI of JavaFX to provide a modern and improved operating experience for users. The default layout features a multi-level menu bar at the top of the window, a shortcut access bar at the bottom, a collapsible toolbox on the left, a home page in the middle, and a meta information and version update record panel on the right (Fig. 2A). The menu bar allows users to quickly launch sub-applications based on multi-level categorization. The Statistics menu includes the Statistics and Algorithm sub-menus, and the Omics menu expands to include the Genomics, Transcriptomics, Proteomics, Metabolomics, Microbial Omics, and Single-cell Omics sub-menus. The Featured menu recommends OmicsSuite exclusive applications such as ImageEditor (Image Editor), PPI-Network, and ShinyEcharts (Shiny based Echarts). The Help menu contains items such as HelpDoc (Help Documents), About, and Update, as well as over 30 tutorial articles that have already been published. The bottom status bar lists quick access links to the source code repository, official website, and tutorials that are convenient for users to access additional content and other analysis services provided by OmicsSuite. The left toolbox’s button interface corresponds to the menu bar items, with a total of 12 categories displayed in a collapsible form that shows all applications. A preview of the resulting image can be seen by hovering the mouse over the button. The thumbnails of all applications are displayed on the home page and remain tied to the functions that launched them. The home page features an introduction to OmicsSuite and a stat chart of all categories based on Apache Echarts (https://echarts.apache.org), which will automatically be updated with new applications. OmicsSuite is continually improved based on feedback from the community, and the version update panel on the right records detailed descriptions of bug fixes, new features, and new applications related to previous versions.

When a sub-application is started, the layout will switch to the user interface, with the analysis page of the application in the middle and application details information (Fig. 2B) on the right. The analysis page is comprised of a data section, a parameter component section, and a result section from top to bottom. The data section allows users to access example data or upload data through a browser button. The conventional tabular data will be displayed in the Table View window, and the rows and columns that can be accommodated can be sorted freely. Specifically, the data section of the sequence analysis application, a Sequence View that allows editing and pasting of FastA or other text types. Both the Table View and Sequence View provide drag-and-drop uploading or copy-and-paste data functions, making it easy for users to verify and edit data after uploading. The parameter component section includes both fixed and variable parameter components. The fixed components Progress, Demo, Clear, and Submit are part of the task management components used to display the current status, run example data, clear the current task, and submit a new task, respectively. Other common components such as Themes, Colors, Fonts, Figure Width, Figure Height, and Figure DPI belong to the parameter specification components. These components implement a unified theme and color scheme for OmicsSuite and standardize the default output image in 10.00 × 6.18-inch (300 dpi) form, following the golden ratio.

According to Java’s explicit data type requirements, the parameter components include a string array type single choice-box, a long text input area, a decimal type slider, an integer type slider, and integrated HSB/RGB/Web color pickers. OmicsSuite strives to provide more parameter interfaces for applications to provide high-quality analysis services and assist users in fully understanding the analysis parameters and testing parameter effects. The result section features a preview of the analysis result image and provides download buttons for PDF, JPEG, TXT, and ZIP file formats, allowing users to download publishable image results and key data files. The right sidebar includes the application name, background, function description, references, and key R package citations.

### Sub-applications overview and classification

**Figure 3:**
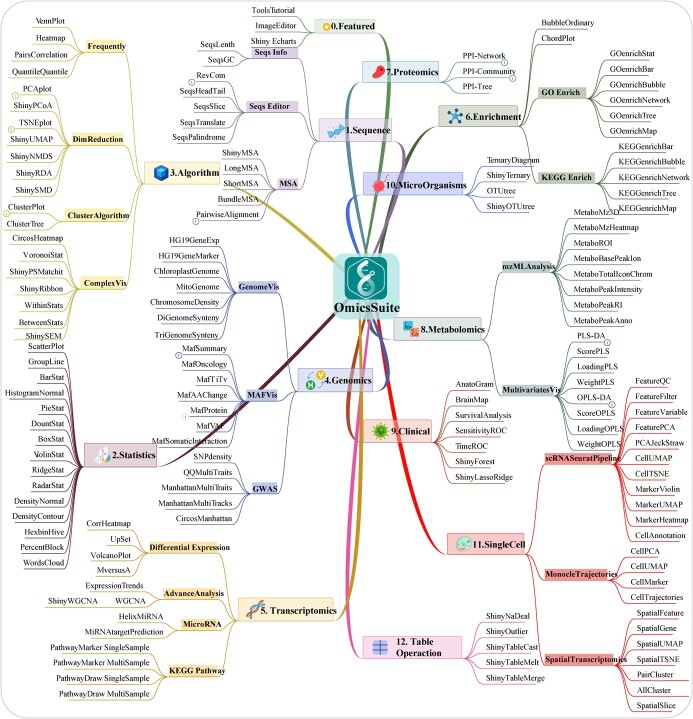
The overview of OmicsSuite 12 categories and 175 sub-applications.

Bioinformatics encompasses biology (such as multi-omics) and methodology (such as statistics and advanced algorithms). Therefore, OmicsSuite continuously improves multi-omics analysis and visualization functions based on the foundation of statistical analysis, providing users with a comprehensive one-stop solution. Currently, there are 12 categories with 175 sub-applications. The categories are: Sequence, Statistics, Algorithm, Genomics, Transcriptomics, Enrichment, Proteomics, Metabolomics, Clinical, Microorganisms, Single Cell, and Table Operation (Fig. 3).

Sequence category in OmicsSuite includes a variety of sub-applications for querying or editing sequences, such as SeqsGC (Sequences GC content) is used for sequences GC content statistics; RevCom (Reverse Complement) performs reverse complement of DNA/RNA sequences (optional only for reverse or complement), and SeqsTranslate (Sequences Translate) realizes the translation of DNA sequences into amino acids (providing genetic codes such as Standard, Vertebrate Mitochondria, and Yeast Mitochondria). In multiple sequence alignment, PairwiseAlignment (Pairwise Alignment) provides five separate alignment methods (Needleman–Wunsch global [31], Smith–Waterman local [32], ends-free overlap, global–local, and local–global) and 10 scoring matrices based on BLOSUM and PAM. The ShinyMSA (Shiny-based Multiple Sequence Alignment) is developed based on the R packages msa [33], msazzR, and bio3d [34] and includes five alignment algorithms [NeighborJoin (Neighbor Joining), UPGMA (Unweighted Pair Group Method with Arithmetic mean), UPGMAMax, etc.] and various sequence coloring schemes (Fig. 3).

OmicsSuite comprehensively refers to the advantages of Excel (Microsoft, Redmond, WA, USA), SPSS (Statistical Package for the Social Sciences, IBM, Armonk, NY, USA), and Prism (GraphPad, San Diego, CA, USA) to develop a customized statistical results and visualization system. The Statistics category includes 15 distinct basic statistical visualization sub-applications. These include ScatterPlot, PieStat, BarStat, BoxStat, PairsCorrelation, RadarStat, DensityNormal, ViolinStat (combined names), etc. The algorithm category integrates algorithms and implements visualization methods such as dimensionality reduction and clustering analyses. We have developed corresponding sub-applications for the dimensionality reduction algorithms PCA (Principal Components Analysis), PCoA (Principal Coordinates Analysis), tSNE (t-distributed Stochastic Neighbor Embedding), UMAP (Uniform Manifold Approximation and Projection), NMDS (Non-metric Multi-Dimensional Scaling) and RDA (Redundancy analysis) that are embedded in PCAplot, ShinyPCoA, TSNEplot, ShinyUMAP, ShinyNMDS, and ShinyRDA (combined names), respectively. All sub-applications draw ellipses with 95% Cis to judge the distance of the scatter plot. Based on the Vega package, R and P values are annotated in the upper-left corner of the figure. Clustering analysis includes seven classic algorithms: Kmeans (K-Means), Hclust (Hierarchical Clustering), AGNES (AGglomerative NESting), Clara, DiAna (Divisive Analysis), Fanny, and PAM (Partitioning Around Medoid). The results of Hclust, AGNES, and DiAna algorithms are suitable for tree-shape; these include rectangle, circular, and phylogenetic shapes. Additionally, we have also originally developed a shinyapp for the SEM (Structural Equation Model) model. ShinySEM (Shiny-based SEM analysis) includes a comprehensive list of functions such as model construction, adjustment, evaluation, optimization, and model frame diagrams visualization (Fig. 3). The categories of multi-omics are described below.

OmicsSuite can analyse almost all multi-omics data, and each classification corresponds to different types of professional data formats. Applications in the OmicsSuite Sequence category typically require a FastA format sequence file, applications in the Genomics category require data in MAF file (Mutation Annotation Format); applications in the Metabolomics category require compressed mzML format files, and users need to provide compressed Matrix or HDF5 format files for the Single-Cell category (Table 1).

**Table 1:** Formats, descriptions, and sample applications for input or output data files

**IO**	**File format**	**Format description**	**Sample app**
Input Format	Text tabulation format	Text tabulation format uses (.txt) as the file suffix and tabs to divide multiple columns of data.	ScatterPlot, BarPlot, BoxStat, etc.
	Sequence FASTA format	Sequence FASTA format uses (.fasta, .fna, .fa) as the file suffix. It’s a text format for representing either nucleotide (DNA/RNA) sequences or amino acid (protein) sequences.	RevCom, LongMSA, SeqsTranslate, etc.
	GenBank data format	GenBank format uses (.genbank, .gb) as the file suffix. Stores information for one or more DNA/RNA/Protein sequences, contains metadata such as annotations, taxonomy.	ChloroplastGenome, MitoGenome
	Mutation Annotation Format	Mutation Annotation Format (MAF) uses (.maf, .maf.gz) as the suffix. Tab-delimited text holds information about genomic mutations, such as SNPs.	MafSummary, MafTiTiv, MafVAF, etc.
	Chromium matrix format	Chromium matrix format uses (.matrix, .mtx) as the file suffix. It contains three files: barcodes.tsv, features.tsv, and matrix.mtx, which are used to store single cell RNA-Seq data.	FeatureQC, PCAJackStraw, CellUMAP, etc.
	Hierarchical Data Format 5	HDF5 uses (.hdf, .h5) as the file suffix. Seurat H5 storages multi-modal single-cell and spatially resolved expression experiments, from CITE-seq or 10X Visium technologies.	SpatialFeature, SpatialGene, SpatialSlice, etc.
	Mass spectrometry mzML	Mass spectrometry mzML format uses (.mzML, .mzML.gz, .mzML.zip) as the file suffix, which stores GC–MS or LC–MS data of proteomics/ metabolomics.	MetaboMzML, MetaboBasePeakIon, MetaboPeakRT, etc.
	Image PDF, JPEG format	Used to store image content.	ImageEditor, SpatialFeature, etc.
Output Format	Text tabulation	Save important intermediate data and results data generated during application analysis.	PCAplot, WGCNA, CellTSNE, etc.
	PDF format	Store the visual output image obtained by the program through analysis, and its size and resolution can be adjusted.	Most OmicsSuite apps.
	JPEG format	Consistent with the PDF results, the default width (10 inch), and height (6.18 inch), The high-quality resolution is 300 dpi.	Most OmicsSuite apps.
	Zip/Gzip compression	For the generated multi-file results, the compressed files are output in Zip/Gzip for convenience.	WGCNA, etc.

With the rapid adoption of bioinformatics, many excellent software or platforms have emerged. We highlight the benefits of OmicsSuite by comparing it to multiple software options (Table 2). Compared to TBtools, SPDE [35], eGPS platform [36], and BioAider [37], OmicsSuite offers the advantages of multi-omics integration, including sequence operations, genome and variation analysis, and transcriptome analysis pipeline. Compared to the Hiplot platform, OmicsSuite offers richer omics data reading functions, proteomics and metabolomics LC–MS (liquid chromatograph mass spectrometer) data analysis pipelines, and a step-by-step customization analysis pipeline of single cell RNA-Seq and spatial transcriptomics. The advantage of online web services is that HPC-based distributed nodes provide powerful computing power, modern parameter components, quiet version updates, access and submission tasks anywhere. Compared with online services, desktop programs are more cost-effective and better suited for small scientific research teams’ maintenance. They provide stable operating status, sufficient analysis tasks and data security, and support local reading of multiple data types instead of uploading. Furthermore, OmicsSuite benefits from the advantages of online services such as user-friendly interfaces, complete parameter components, table and image preview functions.

**Table 2:** Comparison among OmicsSuite and other software or platforms

**Function**	**OmicsSuite**	**Hiplot**	**TBtools**	**SPDE**	**eGPS**	**BioAider**
Software/Service	Software	Service	Software	Software	Service	Software
Plugins 100+	Yes	Yes	Yes	No	No	No
Multi-omics Integration	Yes	Yes	Yes	No	Yes	No
Multi-omics Formats 5+	Yes	Yes	Yes	No	Yes	No
Sequence Operation	Yes	Yes	Yes	Yes	Yes	Yes
Stats Visualization	Yes	Yes	No	No	Yes	No
Genome and Variation	Yes	Yes	Yes	No	Yes	Yes
Transcriptome Pipeline	Yes	Yes	Yes	No	No	No
Proteome Analysis	Yes	No	No	No	No	No
Metabolome Pipeline	Yes	No	No	No	No	No
Mass Spectrometry	Yes	No	No	No	No	No
Single Cell RNA-Seq	Yes	Yes	No	No	No	No
Single Cell Stepping	Yes	No	No	No	No	No
Spatial Transcriptomics	Yes	Yes	No	No	No	No
Clinical Medicine	Yes	Yes	No	No	No	Yes
Microbial Research	Yes	Yes	No	No	No	No
Update Active	High	High	High	Low	Middle	Low

To summarize, the key features of OmicsSuite include: (i) User-friendly interactive experience, convenient demo running button, complete parameter components, and table and image preview windows. (ii) Comprehensive coverage of multi-omics analysis and visualization functions, particularly in metabolomics and single-cell analysis workflows. (iii) OmicsSuite supports reading most multi-omics raw files, such as LC–MS data mzML format, single-cell 10x genomics Chromium matrix format, and Visium HDF5 format data. (iv) OmicsSuite provides a complete basic visualization system, intuitive operation interface for dimensionality reduction algorithms (PCA, PCoA, tSNE, etc.) and clustering algorithms (Kmeans, Hclust, AGNES, etc.), and a SEM model construction and evaluation system.

In less than a year since OmicsSuite was released to the current version 1.3.8, with incomplete statistics from the GitHub release (https://github.com/OmicsSuite/OmicsSuite.github.io/releases), SourceForge stats (https://sourceforge.net/projects/omicssuite-github-io/files/stats/timeline) and Aliyun Drive download channels, OmicsSuite has reached 1000+ downloads. All tutorial articles and videos have received 10 000+ views, and it has received 18 GitHub stars and 23 upvotes from Product Hunt. OmicsSuite will enhance the analysis workflows of multi-omics by long-term development and maintenance.

### Genome, mutation detection, and experimental survival analysis

**Figure 4:**
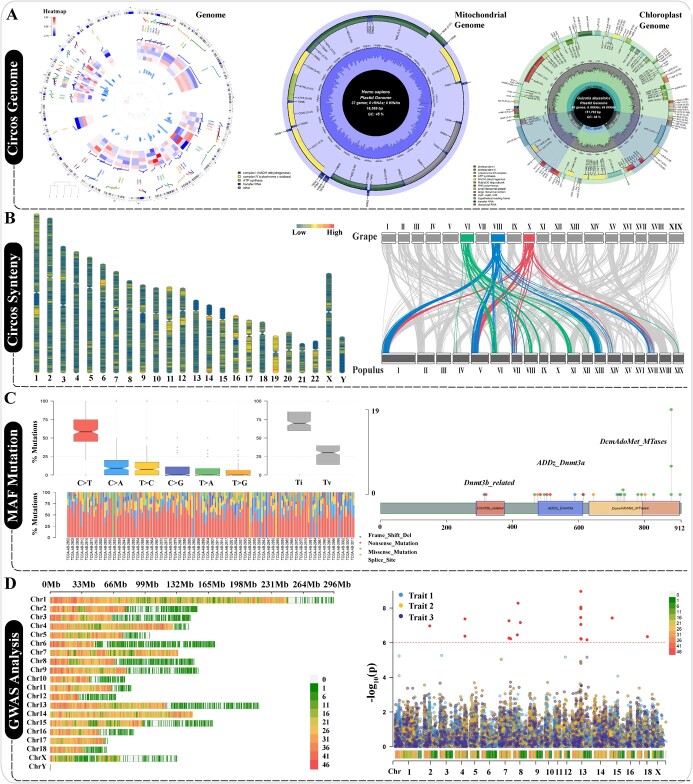
Genome and variation analysis, visualization, and survival analysis. **(A)** Circular plots are used to display the nuclear genome, mitochondrial genome, and chloroplast genome. **(B)** The display mode of the genome at the chromosome level, including columnar haploid and chromosome collinearity. **(C)** Analysis and visualization of variation information contained in MAF files. **(D)** SNP mutation information obtained by GWAS analysis.

The term ‘genome’ refers to the genomes of the nucleus, mitochondria, and chloroplasts. The nuclear genome is used to analyse a species’s DNA sequences in gene and functional annotation, as well as to obtain chromosome structure and other genetic information. This provides reliable reference information for multi-omics [38]. An increasing number of plant and animal genomes are being decoded, and the genomic information at the chromosome level can be visualized in circular or linear representations [39]. GWAS (Genome-Wide Association Study) is widely used to uncover variation sites associated with traits, thereby assisting breeding efforts from a genetic perspective [40]. Genome circle maps can conveniently display gene loci and other information across chromosomes. OmicsSuite has developed four types of genome circle maps based on the Rcircos [41] and chloroplot [42] packages to assist users in visualizing the genome (Fig. 4A). Of these four types, HG19GeneExp (Human Genome v19 Gene Expression) use the built-in human genome version 19 as the reference coordinate system, visualizing gene locus and expression information as scatter plots, bar charts, or heat maps and allowing the marking of specified genes in the results. MitoGenome (Mitochondrial Genome) can parse GenBank-formatted mitochondrial genome information and display the GC content of each gene and of pseudogene markers. The chloroplast genome has more genes than the mitochondrial genome, which encode various tRNAs and rRNAs for protein synthesis, as well as over 50 proteins. ChloroplastGenome (Chloroplast Genome) provides support for displaying the GC content of inverted repeat sequences (IRA/IRB), GC content of genes, pseudogene markers, and InDel information. Users can freely modify the color of each part of the sector using the ColorPicker (Color Picker) component.

**Figure 5:**
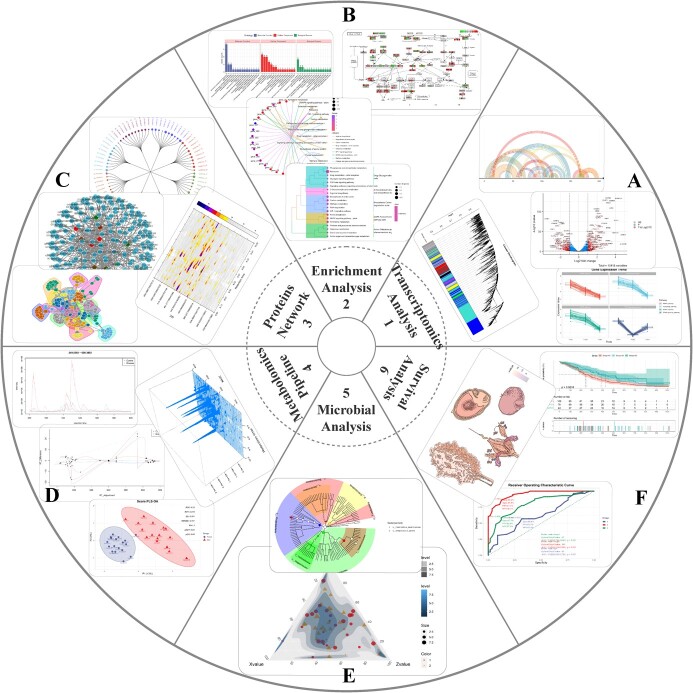
Thumbnail schematic of transcriptomics, enrichment analysis, proteomics, metabolomics, and microorganisms. **(A)** Characteristic analyses in the Transcriptomics category include differential expression analysis, gene expression profiles, and weighted gene co-expression network analysis. **(B)** Characteristic analyses in the Enrichment category. **(C)** Characteristic analyses in the Proteomics category, including PPI network. **(D)** Characteristic analyses in the Metabolomics category, including mzML raw data analysis, Score PLS-DA, and Loading OPLS-DA. **(E)** The OTU phylogenetic tree and relative abundance ternary diagram in the Microorganisms category. **(F)** Survival analysis is used for data analysis of environmental stress or toxicological experiments.

After obtaining a Hi-C chromosome-level genome, chromosome fine mapping and collinearity analysis between chromosomes can be used to analyse the arrangement of genes on chromosomes. In OmicsSuite, ChromosomeDensity (Chromosomes Density) is used to draw chromosomes with centromeres and relative lengths; the function only requires the input of tabled data with chromosome and centromere position information. DiGenomeSynteny (Di-Genomes Synteny) are used to analyse and visualize collinearity among two chromosomes, respectively, methods that are more intuitive when presented in synteny form (Fig. 4B). Genomic variation information can be stored in MAF files, and analysing MAF data is the initial step in analysis of genetic variation. Based on the maftools package [43], OmicsSuite has developed many interactive sub-applications, including MafTiTv (MAF Transitions and Transversions) for analysing statistics of base transitions (Ti) and transversions (Tv), and MafProtein (MAF Protein) for drawing protein domains from the PFAM (Protein Families) database [44] (Fig. 4C). Furthermore, the GWAS strategy identifies SNPs (single-nucleotide polymorphisms) across the entire genome to decipher the potential connection between genetic variation information and phenotype at the chromosome and gene levels. Based on the CMplot (Circle Manhattan Plot) package [45], we have developed customized interactive visualization applications such as SNPdensity (SNP Density) for visualizing the density of variant sites in chromosomes and ManhattanMultiTraits (Manhattan of Multiple Traits), which display multiple groups of SNP variation data in a circular or standard Manhattan form, respectively (Fig. 4D).

### Transcriptomics, protein networks, metabolomics, and microorganism analysis

Gene transcription produces RNAs, including mRNA, miRNA, circRNA, and lncRNA, which contain information concerning gene expression and can be used in transcriptomics research. The tRNA and rRNA are the components of translation [46]. Transcriptomics and metabolomics examine the intrinsic mechanisms of biological processes from the perspectives of gene expression and metabolic compounds, respectively [47, 48]. In the OmicsSuite Transcriptomics category, after sequencing adapters and low-quality base sequences are filtered in FastQ Reads and the gene expression matrix is normalized using RPKM (Reads Per Kilobase per Million mapped reads), FPKM (Fragments Per Kilobase of transcript per Million mapped reads), or TPM (Transcript Per Million), gene differential expression analysis among groups is used as the main theme of transcriptomics analysis. VolcanoPlot and MversusA (MAplot) (combined names) can intuitively display the differentially expressed genes between paired groups. *P*-value or Q-value can be used as the threshold for differences; gene symbols can be added to particularly significant genes, and the color schemes for non-differential, upregulated, and downregulated genes can be customized. ExpressionTrends (Expression Trends) can display the linear trajectories of all genes in multiple groups, making it convenient to observe the expression trends of genes with traits. Similarly, WGCNA performs a weighted gene co-expression network analysis, taking multiple-group gene expression data as input. After a series of processes such as data scaling, soft threshold screening, and module construction, the function finally performs a correlation analysis between modules and traits, presenting the results in heatmaps, and further screens the candidate genes in the significant modules corresponding to each trait. MiRNA negatively regulates mRNA at the post-transcriptional level. We developed MiRNAtargetPrediction (miRNA Target Prediction) and HelixMiRNA (Helix structure of miRNA) to predict the target genes of miRNAs using multiple databases (miRecords [49], miRtarbase [50], miRbase [51]) and to calculate the helix distances of the secondary structure of the miRNAs, respectively (Fig. 5A).

The GO (Gene Ontology) and KEGG (Kyoto Encyclopedia of Genes and Genomes) databases contain a vast amount of genetic information. A gene may be involved in multiple GO terms (cellular component, molecular function, and biological process), as well as KEGG pathways. Annotation and enrichment analyses based on GO and KEGG are crucial for an overview of genes obtained from the genome, transcriptome, and proteome, as well as metabolites obtained from the metabolome. Pathways are often an effective tool for analysing molecular mechanisms. OmicsSuite has developed numerous functions for pathway editing and enrichment analysis based on the pathview [52] and clusterProfiler [53] packages. For instance, PathwayMarkerMultiSample (Pathway of Marker genes of Multiple Samples) can display multiple groups of expression values for the matched genes in the specified pathway, and GOenrichStat (GO Enrichment Statistics) counts GO terms according to the MF, CC, and BP panels of GO and displays the results in a histogram. KEGGenrichNetwork (KEGG Enrichment Network plot) constructs a circular network based on the relationships between genes and pathways, making complex analysis results more understandable. KEGGenrichTree (KEGG Enrichment Tree plot) presents pathways and their secondary classifications in tree form based on enrichment analysis results, and the size of the points indicates the number of genes in the pathway (Fig. 5B).

The proteins analysed in proteomics and metabolites examined in metabolomics are both large molecular weight compounds, and therefore GC/LC–MS platforms are required for ionization and quantitative detection [54]. The resulting raw data is ion mass-to-charge ratio data, which are different from sequencing data that yield sequencing quality values. OmicsSuite offers customized functions PPI-Network, PPI-Community, and PPI-Tree for protein–protein interaction network construction. Users can customize the size, color, and transparency of nodes as well as the layout of the network (tree, circle, or sphere), making it more user-friendly than Cytoscape (Fig. 5C). To integrate the functions provided by MetaboAnalystR [55] and ropls [56] packages, 18 sub-applications have been developed for metabolomics, including exploration of mass spectrometry raw mzML data (from UPLC-QE, HPLC-Q/TOF, HPLC-Ion_Trap, HPLC-Orbitrap, and other platforms) to downstream multivariate analysis. Sample MzML data are filtered based on QCs and then analysed. For instance, MetaboMzHeatmap (Metabolism mzML Heatmap) shows the change in M/Z value with the increase in retention time, MetaboMz3D (Metabolism mzML 3D) shows the M/Z value, retention time, and ionic intensity in a 3D peak graph, and MetaboBasePeakIons (Metabolism Base Peak Ions) shows the curve peaks of ionic intensity with the increase in retention time. We have also redesigned the visualization effects of PLS-DA (Partial Least Squares Discrimination Analysis) and OPLS-DA (Orthogonal Partial Least Squares Discrimination Analysis) based on ggplot2 with functions such as ScorePLS that plots P1 and P2 obtained from score-based PLS-DA analysis in a scatter plot and adds a 95% CI ellipse. Different groups are distinguished by color and shape, and key parameters such as R2X, R2Y, Q2, RMSEE, Pre, pR2Y, and pQ2 are displayed in the upper right corner of the figure. LoadingOPLS has similar basic features to ScorePLS (combined names), except it is based on loading data for OPLS-DA analysis and visualizes results with P1 = 0.0 and P2 = 0.0 axes as references (Fig. 5D).

Microorganisms have the characteristics of being indirectly observable, having small genomes, and being widespread in nature. They have unique strategies for genomic research, including metagenomics and 16S/18S amplicon sequencing. These methods are widely used for microbial community analysis [57]. The OTUtree (Operational Taxonomic Unit evolutionary tree) in OmicsSuite uses the OTU table to construct a microbial phylogenetic tree, and the results are displayed as circle graphs. Users can personalize the colors and layout of different taxonomic units such as Class, Order, and Family. For microbial diversity analysis, TernaryDiagram (Ternary Diagram) can analyse the relative abundances of species in different groups. The coordinate axes represent three experimental groups and the dots represent taxonomic units (e.g. genus). The position of each dot represents the relative abundance value of the genus in different groups (Fig. 5E).

In the Clinical category, AnatoGram (Anatogram) can depict the overall structures or organs of plants or animals, while SurvivalAnalysis (Survival Analysis) is used to analyse the survival status of plants or animals under experimental conditions of drug treatment or environmental tolerance. The analysis results include confidence intervals (Ribbon or Step-style), risk assessment tables, and censoring information. The TimeROC (multiple Time Receiver Operating Characteristic) development can be tailored to support the survival analysis of multiple time nodes in experimental conditions, increasing the efficiency of converting experimental data into research reports (Fig. 5F).

### The pipeline of single-cell RNA-Seq, spatial transcriptomics, and table operations

The application of single-cell sequencing in plants has lagged behind that in animals, but currently, single-cell RNA-Seq and spatial transcriptomics have significant implications in studying plant tissues and cells [58]. Conventional gene expression data are obtained based on the average transcriptome of all types of cells in tissues, while single-cell transcriptome data are more precise and accurate, and biological problems can be solved by associating cell type, gene expression, and phenotypic data [59]. OmicsSuite has developed a pipeline and interactive suite of 22 sub-applications for single-cell transcriptomics based on packages such as Seurat v4 and Monocle2 that provide interfaces for almost all parameters. The 10X Genomics raw data is output in Matrix format after being processed by Chromium, and the results can be read by all sub-applications and entered into the corresponding analysis steps. We recommend providing a minimum of six cores of CPU and 8GB of memory for single-cell analysis, with a test PBMC dataset (including 2700 single cells) execution time of approximately 3 minutes.

**Figure 6:**
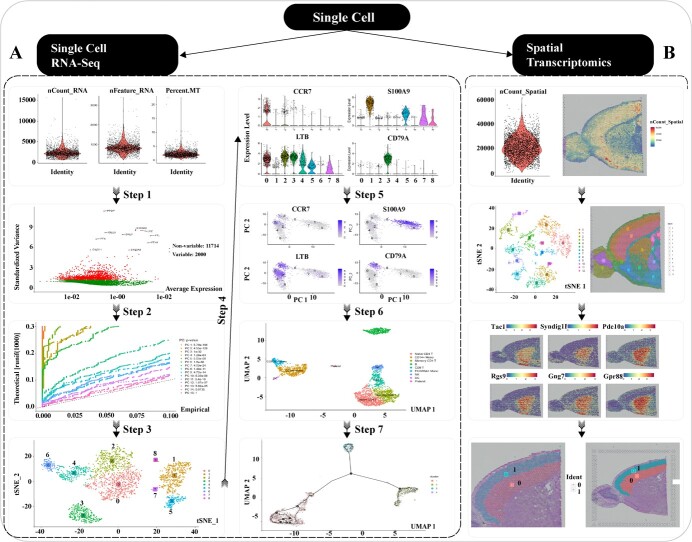
The analysis workflow of single-cell RNA-Seq and spatial transcriptomics. **(A)** The analysis workflow of Single-Cell RNA-Seq. **(B)** The analysis workflow of spatial transcriptomics.

In the single-cell RNA Seq analysis workflow, users can proceed step by step, where PCA table, cell clustering table, and image results are output. FeatureQC (Feature Quality Control) can preliminarily analyse the number of UMIs per cell (nCount_RNA), the number of genes per cell (nFeature_RNA), and the proportion of mitochondrial genes (Percent_MT) per cell. FeatureVariable (Feature Variables) normalizes the data using the LogNormalize (Log Normalize) method and then identifies highly variable genes and outputs the tabulated results. PCAJackStraw (PCA Jackstraw) performs PCA dimensionality reduction analysis on highly variable genes and selects appropriate PCs based on *P*-values calculated by Jackstraw. This step facilitates the outputting of all genes in all PCs and their corresponding *P*-values. CellUMAP and CellTSNE (combined names) use the selected PCs to cluster cells and export table results, and then perform UMAP and TSNE analysis and visualization, respectively. MarkerViolin and MarkerUMAP (combined names) visualize gene markers between cell clusters by screening based on LogFoldChange <0.25 (customizable) and using Violin and UMAP visualization, respectively. Finally, CellAnnotation (Cell type Annotation) annotates cell clusters based on verified cell gene markers and visualizes the UMAP analysis results, as well as MonocleTrajectories (Cell Differentiation Trajectories) analyses and visualizes the cell development trajectories (Fig. 6A).

Utilizing the HDF5 spatial gene expression data and a low-resolution tissue image from 10X Genomics Visium output [28], the programs can perform spatial transcriptomics analysis. Initially, SpatialFeature (Spatial Transcriptomics Feature) displays the correlation between spot molecular counts and cell density in the tissue. SpatialTSNE and SpatialUMAP (combined names) use the SCTransform function to normalize the data, followed by PCA dimension reduction and cell clustering analysis. The results are visualized using TSNE and UMAP methods, respectively, providing downloadable cell clustering table results. SpatialAllClusterMarker (Spatial transcriptomics All Clusters and Marker genes) screens for gene markers that meet the LogFoldChange <0.25 threshold among all obtained cell clusters and exports the qualified data in a Clusters_Markers table and highlights the spots represented by gene markers in the tissue image. Finally, SpacialSlice (Spatial tissue Slice) provides the ability to slice tissue images based on cell clusters (Fig. 6B).

## Conclusion

OmicsSuite comprehensively integrates statistics and multi-omics analysis and visualization. It contains 175 sub-applications in 12 categories, including Sequence, Statistics, Algorithm, Genomics, Transcriptomics, Enrichment, Proteomics, Metabolomics, Clinical, Microorganism, Single Cell, and Table Operation. We created the user interface with Sequence View, Table View, and intelligent components based on JavaFX and the popular Shiny framework. The multi-omics analysis functions were developed based on BioJava and 300+ packages provided by the R CRAN and Bioconductor communities, and it encompasses over 3000 adjustable parameter interfaces. OmicsSuite can directly read multi-omics raw data in FastA, FastQ, MAFMutation Annotation Format, mzML, Matrix, and HDF5 formats, and the programs emphasize data transfer directions and pipeline analysis functions. OmicsSuite can produce pre-publication images and tables, allowing users to focus on biological aspects. OmicsSuite offers multi-omics step-by-step workflows that can be easily applied to horticultural plant breeding and molecular mechanism studies in plants.

## Materials and methods

### Example datasets and data structures

OmicsSuite provides an example dataset for each application to facilitate testing of the functionality and reliability of results during the development process (https://github.com/OmicsSuite/Datasets/). Example datasets can also serve as standard references for user data and can assist users in preparing data with different structures. In OmicsSuite, the parameters represented by all components are provided by Java syntax, which means that Java strict declaration and validation of all variable types. We prefer the TXT text format over the Excel binary format, allowing all functions to be easily read and edited. In typical data table files, standardized data include strings, integers, and decimal types, while consideration is made for NA or special characters (*, −,) that may appear in numeric values. For instance, the first column of the gene expression matrix in WGCNA [60] analysis is labeled as a string type Gene ID; the string type grouping information is provided in the PCA analysis, and only integer or decimal type correlation matrices are allowed in the Pearson correlation heatmap analysis. It is important to note that all example datasets used in applications come from official R packages with built-in datasets, published articles, or publicly available data resources.

### OmicsSuite infrastructure

Java desktop applications are commonly used in software development for their stable performance, powerful functionality, and cross-platform compatibility. Applications such as the IGV (Integrated Genomics Viewer) [61] and the molecular regulatory network visualization program Cytoscape [62] are both available in Java. Java provides a runtime daemon through the JVM (Java Virtual Machine) and implements basic functions such as data type conversion, file transfer, and extraction, encryption and decryption, function and interface, and parameter extraction and processing through core and third-party libraries such as commons-io, commons-compress, and fastjson. OmicsSuite develops a stable desktop suit based on Java and revamps the user interface suitable for data analysis of multi-omics based on JavaFX (https://openjfx.io) framework. R packages have powerful analytical capabilities in the fields of statistics and bioinformatics, and many analytical functions and visualizations are realized by R. These packages are provided by the CRAN (Comprehensive R Archive Network) (https://cran.r-project.org) and Bioconductor (https://www.bioconductor.org) communities, as well as the GitHub (https://github.com) open-source code platform. The GPL-based R scripts are open-sourced and stored in the GitHub repository: https://github.com/OmicsSuite/Rscripts/. OmicsSuite plans to develop new applications in stages and introduce new excellent R packages. OmicsSuite integrates the internal Java runtime environment JRE (v11.0.11) and R runtime (v4.2.2), and thus users can run the software normally without additional configuration after installation.

## Acknowledgements

We thank the CRAN and Bioconductor communities for providing excellent R packages, as well as the developers of R packages used in OmicsSuite. Thanks to Jianming Zeng from University of Macau for his key suggestions in the development process. This work was supported by grants from National Natural Science Foundation of China (32102775), Hainan Province Science and Technology Special Fund (ZDYF2022XDNY234), Earmarked Fund for CARS (No. CARS-49) and Fundamental Research Funds for the Central Universities (2072022). Thanks for the support from the Germplasm resources sharing platform of aquatic species in Fujian Province, XMU-MRB abalone research center.

## Authors’ contributions

B.B.M. and W.W.Y. conceived and designed the project. C.H.K., W.W.Y., X.L., and Z.F.H. provided support and development suggestions for the project. B.B.M. and W.D. wrote the Java, R, Shiny, JavaScript codes for OmicsSuite framework and sub-applications. B.B.M. and Y.X.G. designed the user interface layout and interactive animation. B.B.M. prepared the figures and tables and wrote the manuscript. W.W.Y. reviewed and revised the manuscript. All authors read and approved the final manuscript.

## Data availability

OmicsSuite projects source on GitHub: https://github.com/OmicsSuite/, version release repository: https://github.com/OmicsSuite/OmicsSuite.github.io/, website: https://omicssuite.github.io, R script source codes repository: https://github.com/OmicsSuite/Rscripts/, example datasets repository: https://github.com/OmicsSuite/Datasets/.

## Conflict of interest statement

All authors declare no conflict of interest.
